# Is PET/CT Able to Predict Histology in Thymic Epithelial Tumours? A Narrative Review

**DOI:** 10.3390/diagnostics13010098

**Published:** 2022-12-29

**Authors:** Marco Chiappetta, Paolo Mendogni, Margherita Cattaneo, Jessica Evangelista, Piero Farina, Daniele Antonio Pizzuto, Salvatore Annunziata, Angelo Castello, Maria Teresa Congedo, Diomira Tabacco, Carolina Sassorossi, Massimo Castellani, Mario Nosotti, Stefano Margaritora, Filippo Lococo

**Affiliations:** 1 Department of Translational Medicine and Surgery, Università Cattolica del Sacro Cuore, 00168 Rome, Italy; 2 Thoracic Surgery, Fondazione Policlinico Universitario A. Gemelli IRCCS, 00168 Rome, Italy; 3 Thoracic Surgery, Fondazione IRCCS Ca’ Granda, Ospedale Maggiore Policlnico, 20122 Milan, Italy; 4 Department of Nuclear Medicine, University Hospital Zurich, 8091 Zurich, Switzerland; 5 Unità Di Medicina Nucleare, TracerGLab, Dipartimento Diagnostica Per Immagini, Radioterapia Oncologica ed Ematologia, Fondazione Policlinico A. Gemelli IRCCS, 00168 Rome, Italy; 6 Department of Nuclear Medicine, Fondazione IRCCS Ca’ Granda, Ospedale Maggiore Policlinico, 20122 Milan, Italy

**Keywords:** ^18^F-FDG PET/CT, thymoma, thymic epithelial tumour, radiometabolic assessment, WHO, histology

## Abstract

**Simple Summary:**

Thymic epithelial tumours are rare and insidious malignancies. Histologically, they can be divided into different WHO subtypes and relapse risk classes. Pre-treatment biopsy is not always feasible or accurate in distinguishing WHO classes. ^18^FDG PET/CT scan has been reported to play a remarkable role in the prediction of histology in these tumours (the so-called “non-invasive biopsy”). The present narrative review would like to summarise current evidence on this topic and discuss potential applications.

**Abstract:**

Background: The usefulness of ^18^FDG PET/CT scan in the evaluation of thymic epithelial tumours (TETs) has been reported by several authors, but data are still limited and its application in clinical practice is far from being defined. Methods: We performed a narrative review of pertinent literature in order to clarify the role of ^18^FDG PET/CT in the prediction of TET histology and to discuss clinical implications and future perspectives. Results: There is only little evidence that ^18^FDG PET/CT scan may distinguish thymic hyperplasia from thymic epithelial tumours. On the other hand, it seems to discriminate well thymomas from carcinomas and, even more, to predict the grade of malignancy (WHO classes). To this end, SUVmax and other PET variables (i.e., the ratio between SUVmax and tumour dimensions) have been adopted, with good results. Finally, however promising, the future of PET/CT and theranostics in TETs is far from being defined; more robust analysis of imaging texture on thymic neoplasms, as well as new exploratory studies with “stromal PET tracers,” are ongoing. Conclusions: PET may play a role in predicting histology in TETs and help physicians in the management of these insidious malignancies.

## 1. Introduction

Thymic epithelial tumours (TETs) are rare tumours occurring in the anterior mediastinum, with an estimated incidence of about 1 case per 4 million [1].

The World Health Organization (WHO) histological classification, first issued in 1999 and revised in 2004, is based on morphology and atypia and divides TETs into five types of thymomas (type A, AB, B1, B2 and B3) and thymic carcinomas [2,3]. Many authors have reported that this histological classification represents an independent prognostic factor in patients with TETs [4,5]. In particular, there is a strong body of evidence suggesting that carcinoma has a worse prognosis than thymoma [6]. Taking into account histology and survival outcomes, it is possible to identify a “low-risk class” (including types A, AB and B1 thymomas) and a “high-risk class” (including B2, B3 thymomas and carcinomas) [7]. While this classification is not yet widely accepted, a recent meta-analysis by Marchevsky et al. [8] suggested dividing thymomas into different prognostic subgroups, leaving thymic carcinomas (type C) in a separate class with poorer prognosis.

Surgery represents the mainstay of treatment in patients with TETs and is usually warranted on the sole basis of radiological imaging, without the need for pre-operative biopsy [4]. However, preoperative identification of the histologic subtype could influence the therapeutic strategy; for instance, it could suggest neoadjuvant treatment in patients with locally advanced high-risk TETs [8,9] or rule out minimally invasive surgery (robotic or VATS) in patients with thymic carcinoma [10].

Computed tomography (CT) and magnetic resonance imaging (MRI) are currently used to diagnose mediastinal lesions [11,12], but their ability to differentiate histological subtypes of TETs is limited [13,14]. Over the last decade, interest has emerged in the use of fluorine-18-fluorodeoxyglucose (^18^F-FDG) positron emission tomography (PET) and PET/CT for the evaluation of TETs [15,16]. PET/CT may indeed provide information using not only a qualitative (visual) method but also a semi-quantitative method, such as the calculation of the maximum standardised uptake value (SUVmax). More recently, some authors have even explored the efficacy of ^18^F-FDG-PET-based radiomic and deep-learning features using a machine-learning approach to predict TET histology [17,18].

In this narrative review, the relationship between radiometabolic findings and histological features in TETs is analysed and discussed. Moreover, an overview is provided of the current role and future perspectives of PET/CT in TETs thanks to the availability of new PET tracers and theranostic approaches.

### Methodology

This narrative review is based on a selective literature search carried out in PubMed and Cochrane Library in May 2022. The search string was (“Tomography, Emission-Computed”[Mesh]) AND “Thymus Neoplasms”[Mesh] AND ((humans[Filter]) AND (english[Filter])) + (pet ct AND (thymoma OR thymic carcinoma OR thymic epithelial tumours OR thymic hyperplasia) AND ((humans[Filter]) AND (2021/11/1:2022/5/1[pdat]) AND (english[Filter]))) NOT ((“Tomography, Emission-Computed”[Mesh]) AND “Thymus Neoplasms”[Mesh] AND ((humans[Filter]) AND (english[Filter]))) AND ((humans[Filter]) AND (2021/11/1:2022/5/1[pdat]) AND (english[Filter])) AND ((humans[Filter]) AND (english[Filter])). Overall, our search string identified 193 articles. Two authors (F.L. and P.M.) independently reviewed the abstracts, while a third author (M.Ch.) was consulted in case of discrepancies. Articles were divided into two groups according to whether PET/CT was used to (a) distinguish TETs from thymic hyperplasia or (b) differentiate histology in TETs. Inclusion criteria were: original article, English language, clinical trials (randomised, prospective or retrospective); while exclusion criteria were editorials, letters, case reports, absence of peer review and number of patients included in the series (less than 10 patients for articles on the ability of PET/CT in distinguishing TETs from thymic hyperplasia and less than 20 patients for articles on the ability to differentiate histology in TETs). One hundred and seventy-one articles were excluded after reviewing the abstracts, and a further 5 were excluded following full examination. Finally, 17 articles were suitable for our review: 5 investigated the ability of PET/CT to distinguish TETs from thymic hyperplasia (Table 1) and 12 investigated the ability of PET/CT to differentiate histology in TETs (Table 2). Selected articles were examined in full, processed and summarised according to their relevance and adherence to the topic.

## 2. ^18^F-FDG PET/CT for Predicting Histology in Thymic Epithelial Tumours

### 2.1. PET/CT to Distinguish Thymic Hyperplasia from Thymic Epithelial Tumours

**Figure 1:**
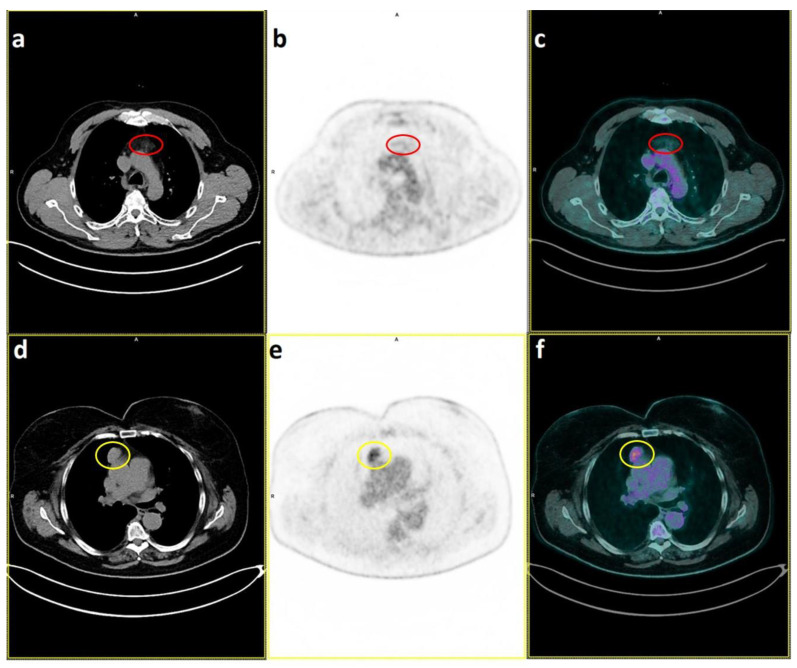
PET CT scan shows absence of uptake in a case of Thymic Hyperplasia ((**a**–**c**), with red circle) [18] compared with mild focused uptake in an A-Thymoma ((**d**–**f**), yellow circle) [36].

Although current guidelines do not recommend pre-operative biopsy in cases of suspected thymoma [36], a distinction between benign conditions (such as hyperplasia) and TETs can alter the therapeutic strategy significantly. Metabolic parameters may prove to be a useful adjunct in the investigation of lesions in the upper anterior mediastinum (Figure 1).

The first report of ^18^F-FDG PET/CT use in TET/hyperplasia was by Liu et al. [19], who evaluated the ratio between SUVmax in the tumour and in the lung (tumour-to-lung ratio or TLR), reporting a significant difference between TETs (TLR: 3.4/3.5) and thymoma (TLR: 5.7 ± 1.7). Other small series analysed SUVmax alone, reporting lower metabolic values in thymic hyperplasia and higher values in TETs. El-Bawab et al. [20] reported SUVmax from 0.7 to 2.5 in hyperplasia compared to 3.1 to 6.1 in thymoma, while Kumar et al. [21] reported an SUVmax of 0.7–1.8 in hyperplasia, 1.7–3.9 in low-risk thymomas and 4.3–9.2 in thymic carcinoma. Moreover, Watanabe and colleagues [22] reported a mean SUVmax of 1.4 ± 0.7 in thymic hyperplasia, 3.7 ± 1.5 in thymoma and 11.4 ± 2.6 in thymic cancer. Again, a significant SUVmax difference was present only between hyperplasia and cancer. Interestingly, in this large series of patients, no case of hyperplastic thymus showed a value of SUVmax higher than 3.

Given the overlapping values, differentiating hyperplasia and low-grade thymomas (A, AB histology) on the sole basis of SUVmax can be challenging. This prompted the study of Travaini et al., who integrated metabolic (i.e., ^18^F-FDG PET/CT) and anatomical (i.e., CT) features [23]. Their study included thymic cysts and found that–despite overlapping SUVmax values in hyperplasia (1.7–5) and low-risk thymomas (2.3–15.5), the integration of anatomical features could help identify 100% of benign lesions. Based on these studies, ^18^F-FDG PET/CT could be an important tool in anterior mass determination and may help differentiate hyperplasia from high-grade thymomas and thymic carcinomas, considering that SUVmax in hyperplasia is rarely higher than 3. However, ^18^F-FDG PET/CT alone cannot discriminate between hyperplasia and low-risk thymomas, to which end morphological evaluation is mandatory, as it could guide differential diagnosis. As a matter of fact, hyperplasia and low-grade thymomas show a distinct CT appearance: V-shape or triangular in hyperplasia compared to nodule/mass in the case of TETs [18]. A further factor to take into account is the spatial distribution of the uptake: low and diffuse across the thymus in hyperplasia, localised in foci or nodules in TETs [18,20].

### 2.2. PET/CT Parameters to Distinguish Histology in TETs

**Figure 2:**
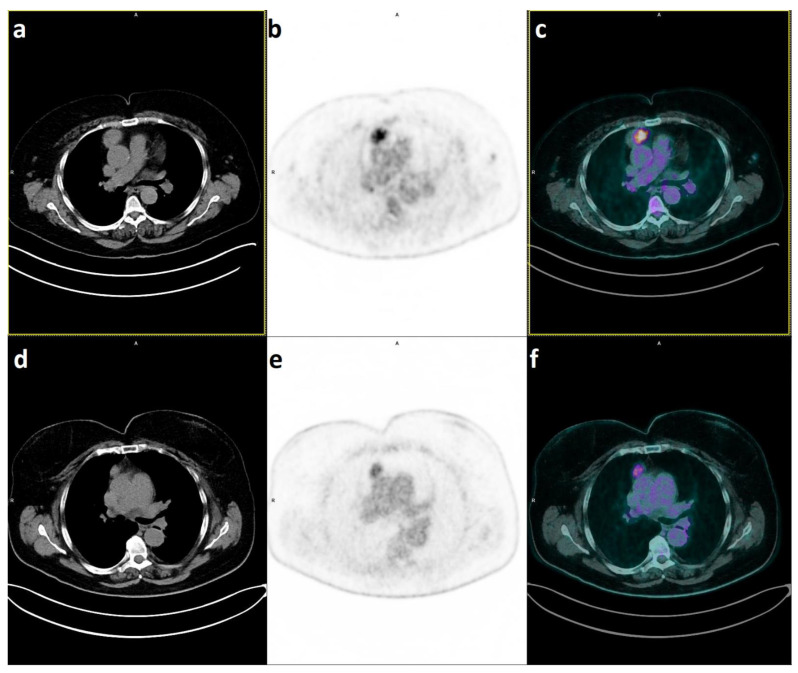
PET CT scan showing the absence of mild uptake in a case of Type AB-Thymoma (**a**–**c**) [18] compared with intense uptake in a Type B3-Thymoma (**d**–**f**) [36].

A simplified histological classification has been proposed to identify different classes of risk in TETs [5]: types A, AB and B1 = “low-risk” thymic neoplasms; B2 and B3 = “high-risk” thymic neoplasms; and thymic carcinoma. The scientific community has largely adopted this simplification and a recent meta-analysis by Marchevsky et al. [6] has confirmed its prognostic value. If ^18^F-FDG PET/CT were confirmed to be able to assess the grade of malignancy in TETs, it could play an important role in the management of the disease (Figure 2). A few studies have shown promising results (see Table 2); most have focused on SUVmax, supporting the use of this metabolic marker in clinical routines [24,26,28,29,31,32,33,34,35,37]. SUVmax has been reported to be consistently higher in carcinoma than in high- or low-risk thymoma, with values between 7.2 and 15.2. In addition to the SUVmax value, the pattern of ^18^F-FDG uptake can provide useful information, as it appears more homogeneous in a higher proportion of thymic carcinomas than thymomas (both low- and high-risk) [24]. A few years ago, our group participated in the first multicentric study on the role of ^18^F-FDG PET/CT as a predictor of WHO classification in a relatively large cohort of TETs (*n* = 47) [16]. SUVmax was found to correlate with WHO malignancy grade (i.e., low vs. high-risk vs. carcinoma), with a Spearman correlation of 0.56 (*p* < 0.0001). Furthermore, we conducted a meta-analysis of 11 studies, which demonstrated a pooled weighted mean difference (WMD) of SUVmax of 1.2 (95%CI: 0.4–2.0) between high-risk and low-risk thymoma, 4.8 (95%CI: 3.4–6.1) between carcinoma and low-risk thymoma and 3.5 (95%CI: 2.7–4.3) between carcinoma and high-risk thymoma [37]. Overall, SUVmax was able to predict histologic subtypes with good accuracy, expressed by an area under the ROC curve ranging from 0.82 to 0.96. Most studies included in the meta-analysis divided TETs into low-risk, high-risk, and carcinoma, except for one that considered only thymoma and carcinoma [14]. In a retrospective study of 51 patients, Benveniste et al. [14] observed significantly higher SUVmax in carcinoma (*n* = 12) and carcinoid (*n* = 2) than in thymoma. SUVpeak and SUVmean also significantly increased in carcinoma.

Readers might have noted that SUVmax values are relatively wide among the abovementioned studies. This could be related to different uptake times, patient obesity, blood glucose levels, different PET/CT scanners or inherent differences among the studied cohorts. In order to overcome these limitations, other metabolic parameters have been proposed. The ratio of SUVmax to tumour size (SUVmax/T) reduces the bias related to tumour dimensions and has been proven to correlate with histologic subtypes of TETs, with an AUC between 0.69 and 0.93 [16,28,32,33]. Similarly, Endo et al. [25] calculated the ratio between SUVpeaks of the tumour and mediastinum (T/M ratio) in 36 patients with histologically proven TETs. Mean T/M ratio differed significantly in low-risk thymoma, high-risk thymoma, and carcinoma (2.64 vs. 4.29 vs. 8.90, respectively, *p* = 0.01).

Volumetric PET/CT parameters, such as metabolic tumour volume (MTV) and total lesion glycolysis (TLG), have been correlated with clinical outcomes in several malignancies. However, their application in TETs showed contrasting results [27,28,35]: in a retrospective monocentric study of 23 patients with pathologically proven TETs (17 low-risk, 6 high-risk, no carcinoma), Bertolaccini and colleagues [27] found that T/M ratio, MTV, and total glycolytic volume (TGV) were able to discriminate between low- and high-risk TETs. Statistical correlation with the WHO classification was higher for TGV (rho = 0.897) than for T/M ratio (rho = 0.873). A TGV cut-off value of 383 seemed to be able to separate low- and high-risk TETs, suggesting its use as a potential parameter in pre-treatment stratification. Volumetric parameters showed higher values in carcinoma than in low- and high-risk thymoma in a retrospective study by Han et al. on 114 patients with TETs [35]. However, Benveniste et al. [14] observed that the total tumour volume (taking into account areas with SUV above 3.5) was larger in thymic carcinoma/carcinoid than in thymoma (*p* = 0.02). The correlation was found only when the total volume was calculated, taking into account areas with SUV above 3.5 (the use of volumes with SUV above 45% of SUVmax failed to show any difference). On the other hand, Park et al. [28] failed to differentiate thymomas and carcinoma on the basis of MTV and TLG. Recently, new approaches have been proposed to predict TET histology by means of 18F-FDG PET/CT. Shinya et al. [30] evaluated metabolic parameters through dual-time-point PET/CT acquisition (i.e., after 90 min and 2 h) in 56 TET patients, suggesting that delayed scanning could improve the diagnostic capacity for high-risk TETs with an accuracy of 82.9% and an AUC of 0.825. A pilot study performed by Ozkan and collaborators in 2022 [38] proposed a machine-learning model and assessed its ability to classify low- and high-risk thymoma on PET/CT images. SUVmax, SUVmean, SUVpeak, MTV and TLG of primary mediastinal lesions were calculated in 27 TET patients. First-, second- and higher-order texture features were also calculated. Among other variables (LDH level and presence of myasthenia gravis), the SHAPE_Sphericity [only for 3D ROI (nz > 1)] was able to differentiate low- and high-risk thymoma.

Despite encouraging results, the integration of these complex parameters into daily clinical practice is far from becoming a reality due to uncertain reproducibility. Therefore, SUVmax remains the most promising parameter for estimating histology in TET patients.

## 3. Future Perspectives

### 3.1. PET Advanced Analysis in Thymic Epithelial Tumours

Advanced imaging analysis, such as radiomics or artificial intelligence applications, could improve the diagnostic and predictive power of PET/CT in thymic tumours and could be used for the prediction of histology and grading.

In a recent paper, Nakajo et al. [17] examined whether a machine-learning approach using ^18^F-FDG PET-based radiomic and deep-learning features could predict the pathological risk subtypes of TETs. Accuracy was significantly higher in the logistic regression model compared to the three SUV-related parameters (i.e., SUVmax, MTV and TLG) for predicting thymic carcinomas, as well as in the random forest model compared to MTV and TLG for predicting high-risk TETs.

The same group previously investigated SUV-related and heterogeneous texture parameters individually and in combination to differentiate between low- and high-risk TETs. The diagnostic performance of individual SUV-related and texture parameters was relatively low. However, combining these parameters could increase diagnostic performance and differentiate between relatively large low- and high-risk TETs [39].

In 2016, Lee et al. found that PET/CT-determined textural heterogeneity indices had the potential to discern between tumour grades, suggesting that these may be integrated with SUVmax in differentiating TET subgroups [40].

Furthermore, larger prospective and validated studies are needed to determine the role of ^18^F-FDG PET/CT radiomics and artificial intelligence applications in thymic tumours, with particular regard to histology and grading prediction.

### 3.2. New “Stromal” Tracers and Other Future Perspectives

No further positron-emitter radiotracer other than ^18^F-FDG has been introduced in the standard workup of patients with thymic neoplasms. However, some cases in the literature described incidental thymic findings during PET/CTs with radiolabelled Choline, ^11^C-acetate and ^68^Ga-PSMA [41].

Quinoline-based PET tracers (which act as fibroblast activation protein [FAP] inhibitors) can detect areas of overexpressed cancer-associated fibroblasts [42]. In this regard, Isik et al. [42] published the case of a 72-year-old woman with metastatic thymic carcinoma referred for salvage peptide receptor radionuclide therapy with ^177^Lu-DOTATATE after completing all treatment options according to current clinical practice guidelines. The patient, however, was not eligible for ^177^Lu-DOTATATE peptide receptor radionuclide therapy and underwent ^68^Ga-FAPI04 PET/CT to assess the potential application of FAP-targeted therapy [39].

Further, larger studies are needed to determine the role of new PET tracers to evaluate the thymic tumour microenvironment, such as radiolabelled FAPI as well as new chemokine receptor ligands (e.g., CXCR4).

## 4. Conclusions

^18^F-FDG PET/CT scan can play a remarkable role in predicting histology in thymic disorders. While there is no robust evidence regarding the ability to differentiate thymic hyperplasia from TETs, it can distinguish carcinoma from thymoma and predict the grade of malignancy (WHO classes) in TETs. In the near future, PET-derived volumetric parameters, texture analysis and new “stromal PET tracers” could help physicians to better characterise and treat thymic lesions.

## Figures and Tables

**Table 1:** PET parameters and thymic pathological findings (hyperplasia vs. tumour) (TLR: tumour to lung ratio).

Study	Year	Patients	Thymic Pathology and Pet-Findings
Liu [19]	1995	12	Thymic hyperplasia: TLR 3.4/3.5 Thymoma: TLR 5.7 ±1.7
El-Bawab [20]	2007	25	Thymic hyperplasia: SUVmax ranging from 0.7 to 2.5 (mean 1.89 ± 0.58) Thymoma: SUVmax ranging from 3.1 to 6.1 (mean 4.75 ± 0.88)
Kumar [21]	2009	23	Thymic hyperplasia: mean SUV max 1.1 (0.7–1.8) Low-risk thymomas: mean SUV max 3 (1.7–3.9), Thymic carcinoma: mean SUVmax 7 (4.3–9.2).
Watanabe [22]	2019	70	Thymic hyperplasia: mean SUVmax 1.4 ± 0.7 Thymoma: mean SUVmax 3.7 ± 1.5 Thymic carcinoid: mean SUVmax 7.0 ± 1.5 Thymic cancer: mean SUVmax 11.4 ± 2.6
Travaini [23]	2008	20	Thymic hyperplasia: SUVmax ranging from 1.7 to 5 Low-grade thymomas: SUVmax ranging from 2.3 to 15.5 High-grade thymomas and thymic carcinomas: SUVmax ranging from 5 to 9

**Table 2:** Relationship between PET/CT findings and TET histology.

Author	Year	Patients	Male/Female	Age	Histology (Number)	PET/CT Parameters	Cut-off Value AUC
Sung [24]	2006	33	15/18	54.6	LR (8) HR (9) CA (16)	SUVmax	NR
						4.0	
						5.6	
						10.5	
Endo [25]	2008	36	21/15	59.1	LR (15) HR (10) CA (11)	T/M SUV	NR
						2.64	
						4.29	
						8.90	
Fukumoto [26]	2012	58	31/27	62	LR (23) HR (21) CA (14)	SUVmax	NR
						3.6	
						4.1	
						7.2	
Lococo [16]	2013	47	25/22	60.9	Thymoma (40) CA (7)	SUVmax	NR
						3.63	0.955
						10.3	
						SUVmax/T	NR
						0.92	0.927
						1.93	
Bertolaccini [27]	2014	23	14/9	52	LR (17) HR (6)	T/M SUV	NR
						1.91 ± 0.45	
						3.73 ± 0.95	
						MTV	NR
						5.51 ± 2.73	
						9.92 ± 2.23	
						TGV	383
						99.12 ± 125.98	
						645.83 ± 159.87	
Benveniste [14]	2014	51	30/21	59.4	Thymoma (37) CA (12) + Carcinoid (2)	SUVmax	NR
						6.27	
						11.09	
						SUVpeak	
						5.53	
						9.38	
						SUVmean	
						3.85	
						6.72	
						TTV_SUV45%	
						176.31	
						153.71	
						TTV_SUV3.5	
						139.29	
						203.01	
Park [28]	2016	61	24/37	50.2	LR (22) HR (32) CA (7)	SUVmax	5.05
						3.43	0.916
						4.42	
						8.23	
						SUVmax/T	NR
						0.65	0.886
						0.91	
						1.77	
						MTV	NR
						90.74	0.512
						80.82	
						90.63	
						TLG	NR
						229.36	0.521
						233.93	
						390.94	
Purandare [29]	2016	52	37/15	49	LR (28) HR (11) CA (13)	SUVmax	6.5
						4.2	0.96
						6.0	
						15.2	
Shinja [30]	2017	56	32/24	NR	LR (27) HR (14) CA (15)	^DTP T/M	2.39
						T/M (early)	
						2.20 ± 0.86	
						2.02 ± 0.77	
						3.57 ± 1.23	
						T/M (delayed)	
						2.29 ± 0.98	2.96
						2.15 ± 0.95	
						3.84 ± 1.55	
Korst [31]	2017	154	37/15	49	LR (74) HR (44) CA (23) others (13)	SUVmax	5.55
						NR	0.79
Tomita [32]	2018	73	37/36	63	LR (41) HR (25) CA (7)	SUVmax	NR
						NR	
						SUVmax/T	NR
						NR	
Zhao [33]	2020	81	43/38	55.6	LR (24) HR (29) CA (28)	SUVmax	5.34
						4.52	0.82
						5.30	
						9.74	
						SUVmax/T	NR
						0.11	0.691
						0.13	
						0.17	
Ito [34]	2021	56	32/24	61.3	LR (26) HR (18) CA (12)	SUVmax	7.40
						4.06	SE 0.84 SP 0.73
						6.01	
						9.09	
Han [35]	2022	114	52/62	56.3	LR (52) HR (33) CA (29)	SUVmax	6.4
						NR	0.94
						MTV	81.3
						NR	0.84
						TLG	117.7
						NR	0.86

LR = Low-Risk; HR = High-Risk; CA = Thymic Carcinoma; T/M SUV = Tumour/Mediastinum SUV ratio; MTV = metabolic tumour volume; TGV = total glycolytic volume; DTP = Dual-Time Point scan; TLG = total lesion glycolysis.

## Data Availability

No new data were created or analyzed in this study.
